# The Hospital Necessities of the Civil Population

**Published:** 1914-08-22

**Authors:** 


### The Hospital

The Workers' Newspaper of Administrative Medicine and Institutional Life, Administration, National Insurance and Health.

No. 1470, Vol. LVI. SATURDAY, AUGUST 22, 1914. PRICE ONE PENNY.

### THE HOSPITAL NECESSITIES OF THE CIVIL POPULATION.

At the present moment, very rightly and naturally, the dangers and risks to which our sailors and soldiers are exposed are prominent in all our minds. We are resolved, each one of us, that, so far as possible, every man broken in the wars shall receive the help which medicine and surgery and skilled nursing can afford him. Every day we read of schemes and societies and subscriptions which express this resolve in action; and, needless to say, we have to such proposals nothing but sympathy and goodwill. We may, however, venture to remind the public that the advent of new claims and duties does not mean that former obligations and demands are cancelled. What is necessary is to add the provision for our soldiers and sailors to the care of the poor who are always with us, and not to substitute the one for the other. Let us do the one by all means, but at the same time let us also be equally careful not to leave the other undone.

These remarks are prompted by the announcement, made on behalf of many of the metropolitan hospitals, that a considerable proportion of the existing hospital beds are to be reserved for sick and wounded sailors and soldiers who may be sent home from the seat of war. For such an arrangement we have the most cordial approval. These men are our first care. Their claim stands in the forefront. They are to have the most efficient help and treatment which can possibly be secured for them. Hence it is seemly that the extensive and efficient equipment of the best modern hospitals should be devoted to their reception and succour. Whatever else may fail in the crisis, this decision must certainly stand secure.

It is necessary, however, to contemplate some of the consequences of such a decision. It is necessary also to meet these consequences. Here is the brief position. Every hospital bed reserved for the sick and wounded involves the subtraction of a bed from the needs of the civil population. The present proposals, speaking of London alone, mean, at a moderate estimate, the reduction of the existing hospital provision for the sick poor by at least 50 per cent. This is to create a most serious position, and we trust that the medical profession will see that it receives due attention and prominence. At any other time, such a proposal would create a profound sensation, and would stimulate to the most vigorous efforts. But amidst the clash of arms there is some danger that it be overlooked.

No one, to put the position quite mildly, will say that the existing hospital facilities in London are in excess of the demand. Seriously to reduce them cannot but mean the exclusion from efficient medical care of patients to whom such care is vital. Further, if, as seems possible, we have before us a period during which there is to be unemployment, there will inevitably be an increase of sickness and suffering, with not less, but greater demand for hospital treatment. Hence the proposed reduction of hospital accommodation takes on a still more serious aspect. The need is likely to increase at the same time as the provision is diminished. Let there be no chance of misunderstanding. We agree most cordially with the proposal to reserve a considerable proportion of the existing hospital accommodation for the benefit of the sick and wounded. But we say that this necessarily means the need of doing something to compensate for the invasion thus made in the supply of hospital help devoted to the needs of the civil population. Unless this is done there will be avoidable suffering and disaster. We wish to escape anything like exaggeration, and we readily allow that some mitigation of the position is possible by the postponement of operations which are not urgent, and by excluding all cases which are not serious and more or less acute. Such steps will lessen the strain, provided this is not long continued. But they certainly will not fully meet a situation in which the supply of hospital beds for the sick poor of the Metropolis is at a single stroke reduced by 50 per cent.

The circumstances here considered throw a great responsibility on the governing bodies of our hospitals. We cannot think they will be unequal to the occasion. By one means or another the difficulty must be met. Some help may possibly be obtained from the Poor-Law infirmaries and by utilising to the utmost the existing hospital accommodation. But there will be instances in which provision must be made by equipping halls or other buildings as hospital wards. Money will certainly be needed, and we urge all those interested in our voluntary hospitals, and all those who ought to be interested, to see that the necessary funds are supplied. To cut a hospital subscription while contributing to one or other of the special funds is neither sacrifice nor patriotism. Some of the horrors of war we must needs support. Let us not add to these the spectacle among our poorer neighbours of injuries and illnesses unrelieved in consequence of deficient hospital accommodation.

